# Disfluent presentations lead to the creation of more false memories

**DOI:** 10.1371/journal.pone.0191735

**Published:** 2018-01-25

**Authors:** Christopher A. Sanchez, Jamie S. Naylor

**Affiliations:** School of Psychological Science, Oregon State University, Corvallis, OR, United States of America; Cardiff University, UNITED KINGDOM

## Abstract

The creation of false memories within the Deese-Roediger-McDermott (DRM) paradigm has been shown to be sensitive to many factors such as task instructions, participant mood, or even presentation modality. However, do other simple perceptual differences also impact performance on the DRM and the creation of false memories? This study explores the potential impact of changes in perceptual disfluency on DRM performance. To test for a potential influence of disfluency on false memory creation, participants viewed lists under either perceptually disfluent conditions or not. Results indicated that disfluency did significantly impact performance in the DRM paradigm; more disfluent presentations significantly increased the recall and recognition of unpresented information, although they did not impact recall or recognition of presented information. Thus, although disfluency did impact performance, disfluency did not produce a positive benefit related to overall task performance. This finding instead suggests that more disfluent presentations can increase the likelihood that false memories are created, and provide little positive performance benefit.

## Introduction

The creation of false memories within the Deese-Roediger-McDermott (DRM; [1]) paradigm is an easily observable, robust, and reproducible effect that has been studied extensively since its initial discovery decades ago [2]. In the DRM and variants, participants first study a list of semantically related words, but due to the highly related nature of the list items, become subsequently more likely to falsely recall and recognize a highly semantically related associate (or lure) that was in fact never presented (see example list in Methods below). The production of false memories within the DRM task has been shown to be sensitive to many factors, on both the individual level (e.g., amount of sleep, mood; [3, 4]), and also related to how these lists of words are presented (modality; [5]) or the instructions given at time of encoding [6]. However, is it possible that other simple perceptual differences in presentation also impact the creation of false memories in the DRM paradigm? For example, other research has suggested that simple changes in the perceptual disfluency of presented material can have significant impacts on judgments related to the task performance, and also may potentially have an influence on how task-relevant information is processed [7]. Thus, an open question is whether disfluency can have a marked effect on the creation of false memories within the DRM task?

### Disfluency and task performance

Research has suggested that perceptually disfluent presentations can negatively impact judgments and/or metacognitive task awareness, although more often than not raw task performance is unaffected by such manipulations [8, 9]. For example, changing text to harder to read fonts, or degrading print quality, not only changes how participants read [7], but also causes participants to rate read information as more difficult, dangerous, or harder to learn [10, 11]. However, in nearly all cases, raw task performance is often spared by disfluency manipulations, such that despite differences in judgment there is often no reduction in memory or learning performance within the task itself [8, 9]. While the effect of disfluency on performance appears neutral in most cases, it has been suggested that in some tasks, specifically those that may benefit from more careful or deliberate processing, disfluency may in fact *increase* task performance [12]. This claim is interesting, as it suggests that simple changes in perceptual characteristics might provide a hidden cognitive benefit towards overall achievement.

A few studies have indeed found a measureable benefit for disfluent presentations, such that tasks that have a large automatic or heuristic component seem to benefit most from such manipulations, via a kind of ‘desirable difficulty’ [13]. For example, [14] found that more disfluent presentations of various simple reasoning tasks caused participants to engage in more systematic and elaborative reasoning, instead of relying on more automatic (and incorrect) heuristics. More disfluent presentations also reduced the likelihood that readers succumbed to misleading semantic distortions by appearing to encourage more word/detail-specific processing in readers (i.e., recognizing the term ‘Moses’ appears in the misleading statement *How many animals did Moses bring on the Ark*?; [8]). Importantly, this performance benefit is not realized in situations where there does not appear to be a large automatic component (i.e., list learning of unrelated items; [9]), further supporting the idea that the attenuation of automatic processes, often through an enhanced item-specific focus, is a key requirement for the facilitative effect of disfluency to emerge [12, 15].

### Disfluency and the DRM

Based on this previous work in disfluency, a task that may be especially sensitive to the effects of disfluency could be a false memory paradigm, specifically the DRM. It has been suggested that the underlying cause of the occurrence of false remembering within the DRM is a tendency to automatically produce holistic relational meaning across studied items [16, 17]. Indeed, several studies have demonstrated that false remembering can be virtually eliminated by reducing automatic processing via enhanced focus on item (or context) specific perceptual features [4, 18, 19, 20, 21]. This expectation is consistent with traditional distinctions between item-specific and relational processing [22], which suggest that a more dedicated focus on item-specific characteristics (and subsequently less relational processing) produces less false recognition [18]. However, it must be noted that other explanations of the false memory effect have also suggested that a more detailed-focus on specific items can potentially *increase* the likelihood of false memory creation via an enhanced automatic spreading of activation brought on by the more in-depth processing [23]. By encouraging a more explicit (and deep) focus on specific items, the semantic associates of said items would be more highly activated passively, thereby increasing the likelihood of recall of these related (but also unpresented) terms. In this case, a more specific item-focus would exacerbate false remembering, rather than reduce it.

While it is not clear whether a more item-specific focus brought on by disfluency would positively or negatively impact the occurrence of false memory, at the very least both of the above explanations suggest that the DRM paradigm should be sensitive to disfluent presentations. As both explanations above rely on an automatic process as a mechanism for increasing or decreasing false remembering (i.e., either a reduction in automatic relational processing, or an increase in spreading activation), any change in the prevalence of that automatic processing should thereby change DRM performance. It has been argued that disfluency manipulations not only produce a reduction in automatic processing in general [14], but likely do so by fostering a heightened perceptual focus on specific items or details [7, 8, 15, 24]. Thus, it seems highly likely that disfluency will impact DRM performance, given the importance of automatic processing relative to this phenomenon.

Further, as the DRM paradigm also allows for the dissociation of correct and incorrect responding, it also potentially affords an opportunity to better understand what might underlie any observed change in performance brought on by disfluency. Previous studies that have demonstrated performance differences as a result of disfluency have so-far only examined correct overall performance (e.g., [8, 14, 24, 25]), without any consideration of errors or mistakes in responding. Given that the DRM task includes assessment of both correct and incorrect responding, an additional unique benefit of exploring disfluency within the DRM is that it provides the opportunity to examine how disfluency impacts responding on multiple levels. Information gleaned from these various task components can potentially clarify not only (1) how disfluency impacts DRM performance, but also (2) how it might impact remembering in a more broad sense. For example, does disfluency simply increase the sheer amount of correct information recalled, or does it instead lead to fewer task errors, or perhaps both? Thus, the findings from a study exploring disfluency within the DRM task have the opportunity to resonate not only within the false memory literature, but also in regards to broader memory research.

Based on previous suggestions related to disfluency, it seems likely that disfluency should foster an item-based focus, which should in-turn affect recall and recognition of information within the DRM. However, it is not clear how this might specifically impact false or incorrect recall, as changes in false recall could be a function of either deeper conceptual processing of specific items, or instead decreased relational processing across items. Regardless, it is anticipated that disfluency should affect DRM performance in some way, given the potential for multiple interactions with various automatic processes that have been suggested to underlie false memory formation.

## Method

### Participants

Previous investigations using the same font disfluency manipulation utilized here [7, 8] suggest an average effect size of approximately *d* = .92 on relevant measures. Results from an *a priori* power analyses with a β = .80, thus recommended a minimum of approximately 20 participants in each condition. To this end, 54 native English speaking participants were recruited for the experiment, however 3 participants were omitted from final analyses as they accurately identified the nature of the experiment at post-test (see below). Thus, *N* = 51 students (39% female) from an undergraduate Psychology course at a large public university successfully participated in the experiment for course credit. This study was officially approved by the Oregon State University Institutional Review Board prior to its conduct.

### Materials

#### DRM false memory task, recall & recognition

Participants were asked to view and remember 6 lists drawn directly from [1]. These lists were 15 words long, and participants were given 30 seconds to study each list, consistent with the procedure of [1]. An example list would be: *nurse*, *sick*, *lawyer*, *medicine*, *health*, *hospital*, *dentist*, *physician*, *ill*, *patient*, *office*, *stethoscope*, *surgeon*, *clinic*, *cure;* unpresented associate lure: *doctor*. The order in which participants viewed the 6 lists was randomized across participants, however items within lists were always presented in identical order for all participants. Importantly, participants were randomly assigned to either a fluent (*n* = 25) or disfluent (*n* = 26) presentation. In the disfluent condition, participants viewed all lists of words in 14 pt. Mistral font, while the fluent condition instead viewed all lists in 14 pt. Arial font. This font manipulation has been shown previously to increase estimates of perceptual disfluency [7, 8].

After the study time for a given list had elapsed, participants were asked to recall all the words they could from that list, in any order. Participants were then shown the next list, and asked to recall it, and so on, until they had viewed and recalled all 6 lists. Recall lists were scored for (1) total number of words recalled (both correct and overall), and (2) the accuracy of recall (i.e., # of correctly recalled words which were present on the list/total # of words recalled). Finally, lists were also evaluated for the (3) recall of the critical conceptual lure, and the number of times this associate lure recall occurred was then aggregated over the 6 lists (maximum score of 6).

Participants were also asked to complete a 48-item recognition test. This test was comprised of 24 ‘old’ items (i.e., 4 items from each of the presented 6 lists), 18 ‘new’ items (i.e., items not presented on the previous lists, but instead drawn from other unrelated lists from [1]), and finally the 6 critical associates (i.e., items not presented, but semantically related to the presented lists). The order of individual items on the recognition test was randomized across participants, and font was matched to the original encoding conditions to eliminate any influence of context dependent recognition factors. Participants were asked to rate these items on a 1–4 scale (1 = *Sure was not studied*, 2 = *Probably was not studied*, 3 = *Probably Studied*, 4 = *Sure was studied*). Higher ratings are indicative of higher perceived familiarity.

#### Task difficulty ratings

Consistent with other prior investigations of perceptual fluency, participants were asked to provide ratings on a 1–10 scale about (1) *How difficult was the overall task*?, and (2), *How hard was it to read the words*?. These measures served 2 purposes: to serve as an experimental check for the disfluency manipulation, and also provide an estimate of whether this difference in disfluency influenced judgment of task difficulty.

#### Working memory capacity (WMC)

To control for general cognitive ability, which has been shown to impact memory recall in various contexts including false memory paradigms [26], all participants completed a computerized WMC task (Symmetry Span, SSpan; [27]). In this complex span task, for every trial participants were asked to make a symmetry judgment along a vertical axis, after which they were then shown a spatial location in a 4x4 matrix to remember for later recall. Trials were grouped into sets of 3–5 trials, and participants saw each set 2 times. This task has been previously shown to be an accurate and reliable predictor of WMC, at levels consistent with other complex span tasks [27, 28].

### Procedure

After completing informed consent, participants were randomly assigned to either the fluent or disfluent presentation conditions. Participants then were presented the 6 word lists, and asked to recall each list after it was presented. After viewing and recalling all 6 lists, they then completed the Task Difficulty Ratings. Participants then completed a 1 minute filler task in which they solved simple math problems (e.g., *1 x (7–5) =*?) to ensure they were not actively rehearsing any presented lists, after which they then completed the Recognition test, and finally the WMC task. Participants were then asked to guess what the experiment was about, and if participants in any way suggested that the task was designed to examine false memory, they were not retained for further analysis. Participants were then debriefed and dismissed. The entire experiment took no longer than 1 hour.

## Results and discussion

Descriptive statistics for all measures are available in Table 1.

**Table 1:** Descriptive and inferential statistics by disfluency condition.

	Fluent (*n* = 25)	Disfluent (*n* = 26)	*t*(49)	*p*	Cohen’s *d*
Symmetry Span	15.56(3.78)	13.85(4.83)	1.41	.17	.39
Task Difficulty					
How hard to read?	2.04(2.49)	6.85(2.29)	7.17*	.00	2.01
How hard to recall?	5.44(1.69)	6.42(1.55)	2.17*	.04	.60
Recall					
Total words recalled on average	9.37(1.87)	8.95(1.33)	.92	.36	.26
Recall accuracy (%)	98(1)	96(2)	4.26*	.00	1.20
Avg. # correct words recall	9.19(1.77)	8.58(1.28)	1.42	.16	.39
Frequency lure recall	.84(.69)	1.69(1.23)	3.05*	.004	.86
Recognition (1–4)					
Familiarity ratings of seen words	3.41(.36)	3.44(.31)	-.36	.72	.09
Familiarity ratings of new words	1.45(.37)	1.54(.45)	-.79	.43	.22
Familiarity ratings of lures	2.88(.56)	3.22(.53)	2.20*	.03	.62

*p < .05

Task difficulty and reading difficulty ratings were completed on a 1–10 scale, with 1 being lowest. Recognition familiarity ratings were on a 1–4 scale (1 = *Sure was not studied*, 2 = *Probably was not studied*, 3 = *Probably Studied*, 4 = *Sure was studied*).

### Working memory capacity and task difficulty

Importantly, there was found to be no reliable difference between disfluency groups in performance on the WMC task (*t*(49) = 1.41, *p* = .17), suggesting that the groups were well-matched on general cognitive ability.

However, consistent with prior research on disfluency, participants in the disfluent condition rated the text as significantly harder to read (*t*(49) = 7.17, *d* = 2.01, *p* < .001), and also rated the recall task as significantly more difficult to complete (*t*(49) = 2.17, *d* = .60, *p* = .04).

### List recall and recognition

In terms of the sheer amount of words recalled on average, there was no reliable difference between presentation conditions (*t*(49) = .92, *p* = .36). However, in terms of the accuracy of the words recalled, while the information both groups recalled was mostly correct (> 95% correct), there was a significant difference in accuracy between conditions, with the fluent condition performing at a significantly higher level than the disfluent condition (*t*(49) = 4.26, *d* = 1.20, *p* < .001). This difference in accuracy was likely driven by the higher rate of associate lure recall in the disfluent condition (*t*(49) = 3.05, *d* = .86, *p* = .004), as there was no reliable difference in the average number of correct words recalled across conditions (*t*(49) = 1.42, *p* = .16). Thus, while there was still evidence of false remembering in the fluent condition (significantly > 0; *t*(24) = 6.11; *p* < .001), reliably more false recall occurred in the disfluent condition. This pattern of effects in interesting, as it suggests that while the overall amount of appropriate information participants output (i.e., the raw number of correct words recalled) is unaffected by the disfluency manipulation, disfluency does increase the recall of erroneous information (i.e., the critical lures).

This pattern of effects was exactly mirrored on the recognition test. There was no reliable difference in the ratings of either previously seen, or never seen, words across disfluency conditions (*t*s < .79, *p*s>.43). However, there was a significant difference in recognition ratings for the critical lures (*t*(49) = 2.20, *d* = .62, *p* = .03), such that those participants in the disfluent group rated the lures as *more* likely to have been presented on the previous lists. Consistent with the recall results above, it appears that correct recognition of explicitly presented/unpresented information remains unaffected, however disfluency does appear to increase recognition of semantically related information that was never presented.

## Conclusions

The goal of the current study was to examine whether changes in perceptual disfluency significantly impacted performance in the DRM task, and the creation of false memories. As it has been suggested that this false memory phenomenon is often a result of various automatic processes, it was speculated that disfluent presentations should change false remembering by encouraging an enhanced perceptual item focus, which has been suggested to curtail automatic processing. While the classic disfluency effect was replicated, such that participants rated the task as more difficult and *correct* recall and recognition were unaffected (either positively or negatively), it does appear that manipulation of perceptual disfluency in fact made participants more likely to make semantic *errors* of both recall and recognition. While participants in both conditions were generally highly accurate in what information they did recall, even at these high levels of accuracy participants in the disfluent condition were less so, due to the more frequent recall of semantic lures. In other words, disfluent presentations not only caused participants to rate task performance as more difficult, but also significantly increased the likelihood of generating errors of false recall and recognition within the DRM task. Thus, while the current study does demonstrate that disfluency can change performance, these latter findings stand in direct contrast to previous suggestions for an overall disfluency benefit [14, 25]. This finding is especially troubling as it suggests that not only does disfluency not increase correct task performance as suggested elsewhere, but in fact increases the likelihood of making task errors, in this case false associations. Further, as disfluency does seem to specifically affect task errors, it is reasonable to expect that in even more difficult tasks this pattern of effects may become even more pronounced, but this remains to be verified.

The results of this study also highlight an interesting issue regarding the creation and prevalence of false memories. As mis-remembering appears to occur even under perceptually normal conditions (and was observed here even in the perceptually fluent condition), under less perceptually salient conditions the likelihood of creating false memories appears to increase by roughly a factor of two. This could be especially problematic for situations that rely heavily on the veracity of reported information (e.g., eyewitness testimony) as it suggests that when perceptual conditions are less than optimal, individuals are even *more* likely to falsely remember information that never actually was present. This effect is especially sobering given the frequency of violent criminal events, for example, that occur more often during perceptually degraded conditions (i.e., dusk, night-time; [29]). Whether or not this finding is replicated in these more applied settings remains an empirical question, however, the current results do seem to strongly support a potential interaction between disfluency and false memory creation.

In conclusion, this study does find that disfluency can impact performance on the DRM, but in ways that are perhaps non-optimal relative to overall task performance. Disfluency did not increase overall correct recall or recognition, and in fact increased incorrect responding in both these tasks. Thus, disfluency does seem to significantly increase the likelihood of creating or recognizing false associates, while simultaneously not providing any positive benefit towards recall or recognition. This is potentially a very important addendum to the false memory literature, as in real world settings that are often not perceptually optimal, it is possible that the presence of any perceived disfluency (whether environmental or not) may lead to higher rates of false memory creation. In a larger sense, these findings are also consistent with a growing body of research that has also failed to demonstrate a positive benefit for disfluency (e.g., [30, 31]). As a whole, these results provide an additional data point in terms of understanding not only how disfluency may impact performance, and under what contexts, but also suggests that there are other additional perceptual factors that may impact the creation of false memories within the DRM.

## References

[1] Roediger HL, McDermott KB. Creating false memories: Remembering words not presented in lists. Jour of exp psych: Learn, Mem, and Cog. 1995;21:803–814.

[2] Deese J. On the prediction of occurrence of particular verbal intrusions in immediate recall. Journal of exp psych. 1959;58:17–22. 10.1037/h0046671 13664879

[3] Payne JD, Schacter DL, Propper RE, Huang LW, Wamsley EJ, Tucker MA, Walker MP, Stickgold R. The role of sleep in false memory formation. Neurobiology of learn and mem. 2009;92:327–34. 10.1016/j.nlm.2009.03.007 PMC2789473 19348959

[4] Storbeck J, Clore GL. With sadness comes accuracy; with happiness, false memory: Mood and the false memory effect. Psych Sci. 2005;16:785–91. 10.1111/j.1467-9280.2005.01615.x 16181441

[5] Smith RE, Hunt RR. Presentation modality affects false memory. Psycho Bull & Rev. 1998;5:710–15.

[6] Read JD. From a passing thought to a false memory in 2 minutes: Confusing real and illusory events. Psycho Bull & Rev. 1996;3:105–11. 10.3758/BF03210749 24214811

[7] Sanchez C. A., & Jaeger A. J. If it’s hard to read, it changes how long you do it: Reading time as an explanation for perceptual fluency effects on judgment. Psych Bull & rev. 2015; 22: 206–11. 10.3758/s13423-014-0658-6 24825308

[8] Song H., & Schwarz N. Fluency and the detection of misleading questions: Low processing fluency attenuates the Moses illusion. Soc Cog. 2008; 26: 791–99.

[9] Yue C. L., Castel A. D., & Bjork R. A. When disfluency is—and is not—a desirable difficulty: The influence of typeface clarity on metacognitive judgments and memory. Mem & Cog. 2013; 41: 229–41. 10.3758/s13421-012-0255-8 22976883

[10] Rhodes M. G., & Castel A. D. Memory predictions are influenced by perceptual information: evidence for metacognitive illusions. Journ Exp Psych: Gen. 2008; 137: 615–25. 10.1037/a0013684 18999356

[11] Winkielman P., Schwarz N., Fazendeiro T., & Reber R. The hedonic marking of processing fluency: Implications for evaluative judgment 2003; In Musch J. & Klauer K. C. (Eds.), The psychology of evaluation: Affective processes in cognition and emotion (pp. 189–217). Mahwah, NJ: Erlbaum.

[12] Oppenheimer D. M. The secret life of fluency. Trends in Cog Sci. 2008; 12: 237–41. 10.1016/j.tics.2008.02.014 18468944

[13] Bjork R. A. Memory and metamemory considerations in the training of human beings 1994; In Metcalfe J. & Shimamura A. (Eds.), Metacognition: Knowing about knowing (pp. 185–205). Cambridge, MA: MIT Press.

[14] Alter A. L., Oppenheimer D. M., Epley N., & Eyre R. N. Overcoming intuition: metacognitive difficulty activates analytic reasoning. Journ Exp Psych: Gen. 2007; 136, 569–576. 10.1037/0096-3445.136.4.569 17999571

[15] Schwarz N., & Clore G. L. Feelings and phenomenal experiences 2007; In Kruglanski A. & Higgins E. T. (eds.), Social psychology. Handbook of basic principles (2nd ed.; pp.385–407). New York: Guilford.

[16] McDermott KB. Priming on perceptual implicit memory tests can be achieved through presentation of associates. Psych Bull & Rev. 1997; 4:582–6.

[17] McKone E., & Murphy B. Implicit false memory: Effects of modality and multiple study presentations on long-lived semantic priming. Jour of Mem and Lang. 2000; 43: 89–109.

[18] Arndt J, Reder LM. The effect of distinctive visual information on false recognition. Jour of Mem and Lang. 2003; 48:1–5.

[19] Hege AC, Dodson CS. Why distinctive information reduces false memories: evidence for both impoverished relational-encoding and distinctiveness heuristic accounts. Jour of Exp Psych: Learn, Mem, and Cog. 2004; 30:787. 10.1037/0278-7393.30.4.787 15238023

[20] Mccabe DP, Presmanes AG, Robertson CL, Smith AD. Item-specific processing reduces false memories. Psych Bull & Rev. 2004; 11:1074–9. 10.3758/bf03196739 15875978

[21] Whittlesea BW. False memory and the discrepancy-attribution hypothesis: the prototype-familiarity illusion. Jour of Exp Psych: Gen. 2002;131:96. 10.1037//0096-3445.131.1.96 11902153

[22] Hunt RR, Einstein GO. Relational and item-specific information in memory. Jour of Verb Learn and Verb Behav. 1981; 20:497–514.

[23] Loftus EF. Memories of things unseen. Curr Dir in Psych Sci. 2004; 13:145–7.

[24] Diemand-Yauman C., Oppenheimer D. M., & Vaughan E. B. Fortune favors the (): Effects of disfluency on educational outcomes. Cog. 2011; 118: 111–5. 10.1016/j.cognition.2010.09.012 21040910

[25] Weissgerber SC, Reinhard MA. Is disfluency desirable for learning?. Learn and Instruct. 2017; 49:199–217.

[26] Peters MJ, Jelicic M, Verbeek H, Merckelbach H. Poor working memory predicts false memories. Euro Jour of Cog Psych. 2007; 19:213–32.

[27] Oswald FL, McAbee ST, Redick TS, Hambrick DZ. The development of a short domain-general measure of working memory capacity. Behav Res Methods. 2015;47:1343–55. doi: 10.3758/s13428-014-0543-2 25479734 10.3758/s13428-014-0543-2

[28] Kane MJ, Hambrick DZ, Tuholski SW, Wilhelm O, Payne TW, Engle RW. The generality of working memory capacity: a latent-variable approach to verbal and visuospatial memory span and reasoning. Jour of Exp Psych: Gen. 2004; 133: 189–217 10.1037/0096-3445.133.2.189 15149250

[29] Felson M, Poulsen E. Simple indicators of crime by time of day. Int Jour of Forecasting. 2003; 19: 595–601.

[30] Thompson V. A., Turner J. A. P., Pennycook G., Ball L. J., Brack H., Ophir Y., & Ackerman R. The role of answer fluency and perceptual fluency as metacognitive cues for initiating analytic thinking. Cog. 2013; 128, 237–51. 10.1016/j.cognition.2012.09.012 23158572

[31] Meyer A., Frederick S., Burnham T. C., Guevara Pinto J. D., Boyer T. W., Ball L. J., et al Disfluent fonts don’t help people solve math problems. Journ Exp Psych: Gen. 2015; 144, e16–e30. 10.1037/xge0000049 25844628

